# YouTube Searching and Self-Treatment Behaviors Among Patients With Benign Paroxysmal Positional Vertigo Before and After Clinic Visits: Prospective Observational Study

**DOI:** 10.2196/80966

**Published:** 2025-12-01

**Authors:** Guil Rhim, Moon Jung Kim

**Affiliations:** 1 Department of Otorhinolaryngology, One Dizziness Research Center, One Otorhinolaryngology Clinic, Paju, Republic of Korea; 2 Department of Laboratory Medicine, Myunggok Medical Research Center, Konyang University College of Medicine, 158 Gwanjeodong-ro, Daejeon, 35365, Republic of Korea, 82 426009307

**Keywords:** YouTube, health information, benign paroxysmal positional vertigo, behavior, self-treatment

## Abstract

**Background:**

YouTube has become a popular platform for patients seeking health-related information, including guidance on managing benign paroxysmal positional vertigo (BPPV). As self-diagnosis and self-treatment through online content grow more common, concerns have arisen regarding their influence on patients’ health care decisions and treatment outcomes. However, little is known about how YouTube use and self-treatment behaviors change before and after clinical consultation, or whether these behaviors affect standard care for BPPV.

**Objective:**

This study aimed to investigate changes in patients’ YouTube searching and self-treatment behaviors before and after clinic visits for BPPV and to assess whether self-treatment influences standard in-clinic management.

**Methods:**

A prospective study was conducted with patients diagnosed with BPPV who visited an otorhinolaryngology clinic in Korea from August 2024 to July 2025. On the final day of treatment, participants completed a survey, and chart reviews were performed to collect data on age, sex, canal involvement, chronic disease status, number of canalith repositioning maneuver (CRM) sessions, and pre- and postclinic YouTube searching and self-treatment. Differences in pre- and postclinic behaviors by gender were analyzed using Generalized Estimating Equations for repeated measures. The effect of self-treatment on the number of CRM sessions was assessed using negative binomial regression after confirming overdispersion.

**Results:**

Among 147 patients (71% women), preclinic YouTube searching was reported by 28 (25%) patients, while postclinic searching decreased to 21 (14%) patients. Gender-stratified Generalized Estimating Equations analysis showed women had significantly higher odds of preclinic YouTube searching compared to postclinic (odds ratio [OR] 2.389, 95% CI 1.195‐4.778, *P*=.01). Additionally, women with chronic disease had significantly lower odds of self-treatment (OR 0.13, 95% CI 0.016‐0.976, *P*=.047). Negative binomial regression showed no significant association between self-treatment status and the number of CRM sessions.

**Conclusions:**

This study demonstrates that YouTube searching and self-treatment behaviors for BPPV change following clinical consultation. These findings highlight the importance of patient education during clinical encounters in addressing previsit online information use and mitigating inappropriate self-treatment practices.

## Introduction

The influence of social media on health, public health, and disease management through information searching and acquisition is increasing [1]. In particular, the use of YouTube for obtaining health-related information is steadily rising, and its impact on health is becoming increasingly significant [2]. As a video-sharing platform, YouTube provides viewers with tools that facilitate the easy replication of behaviors observed in videos. Benign paroxysmal positional vertigo (BPPV) is a condition characterized by the displacement of otoconia from the maculae of the utricle into one or more of the three semicircular canals or their adhesion to the cupula, causing clinical symptoms due to positional changes [3]. The diagnosis of BPPV is based on identifying characteristic nystagmus, along with a thorough history taking and physical examination, using methods such as the Dix-Hallpike test and the head roll test [4,5]. The standard and highly effective treatment for BPPV is the canalith repositioning maneuver (CRM), with success rates exceeding 80% when the diagnosis is accurate [6].

The first study evaluating YouTube video content related to BPPV was published in 2012 [7]. Since then, only a few additional studies have examined BPPV-related YouTube content, and most of these have primarily focused on evaluating the validity of the information. While YouTube contains information on almost all diseases, numerous studies have assessed the reliability and accuracy of this content across various medical topics.

What distinguishes BPPV from other conditions is that YouTube hosts video content specifically on CRM, the standard treatment for BPPV, which patients can follow to potentially achieve direct therapeutic effects. In Korea, BPPV is also the most common cause of dizziness [8], and a significant amount of information about BPPV is available through YouTube searches. People in Korea often search for BPPV using Korean-language terms when looking for information online.

In our recent clinical practice, we have observed an increasing number of patients with dizziness symptoms who report searching for BPPV information on YouTube or attempting self-treatment before visiting a hospital. While previous studies focused on evaluating YouTube content from an expert perspective, this study adopts a user-centered approach to explore how patients experience and use BPPV-related information on YouTube. Specifically, it aims to examine how YouTube searching and self-treatment behaviors change before and after clinic visits, and to assess whether self-treatment influences standard in-clinic management for BPPV.

## Methods

### Study Design

This prospective study was conducted with patients diagnosed with BPPV who visited the otorhinolaryngology clinic due to vertigo symptoms from August 2024 to July 2025. A total of 149 patients met the eligibility criteria, and 147 of them were included in the final analysis. When a patient presented with vertigo symptoms and abnormal findings were noted during medical history taking, general otolaryngological examination, and physical examination using Frenzel glasses, videonystagmography tests were performed. After a diagnosis of BPPV was confirmed, CRMs were performed once per session, typically 2‐3 times per week. The specific maneuver was selected according to the involved canal: the modified Epley method was used for canalolithiasis of the posterior semicircular canal, the Barbecue rotation method was used for canalolithiasis of the lateral semicircular canal, and the Appiani and Gufoni methods were used for lateral-canal cupulolithiasis. In cases of multicanal involvement, only one canal was treated per session. On the final day of treatment, a survey was administered, and patient chart reviews were conducted to collect information on sex, age, number of CRM sessions, chronic disease status (hypertension or diabetes), canal involvement, preclinic YouTube searching, preclinic self-treatment, YouTube searching during treatment (postclinic), and self-treatment during treatment.

### Ethical Considerations

This study was approved by the Korea National Institute for Bioethics Policy (P01-202408-01-036). All patients gave their informed consent prior to their inclusion in the study, and this study has been performed in accordance with the ethical standards in the 1964 Declaration of Helsinki and its later amendments. Data collection was conducted in July 2025 for research purposes. All data were fully anonymized prior to analysis, and the authors had no access to personally identifiable information. Participants did not receive any financial or nonfinancial compensation for their participation in this study.

### Statistical Analysis

Descriptive statistics were used to summarize the included studies by author, origin country, publication type, research focus, and primary topic area. Differences in pre- and postclinic YouTube searching and self-treatment behavior by gender were analyzed using Generalized Estimating Equations (GEE) for repeated measures. To assess the effect of self-treatment on the number of CRM procedures, Poisson regression was initially applied, but overdispersion was confirmed (Value/df=372), leading to the use of a negative binomial regression model. A *P* value of <0.05 was considered statistically significant. All analyses were conducted using SPSS software (version 19.0; IBM Corp).

## Results

A total of 147 patients were included in the analysis, comprising 104 (71%) women and 43 (29%) men. The age of women ranged from 17 to 86 years (mean 55.6, SD 13.3), while that of men ranged from 11 to 80 years (mean 52.7, SD 15.5). Among the 147 patients, regarding canal involvement, 18 patients (12%) had posterior canal BPPV, 56 (38%) had lateral canal involvement, 41 (28%) had multiple canal involvement, and 32 (22%) had combined canal and cupulolithiasis involvement. A history of hypertension or diabetes was present in 53/147 patients (36%).

Until the end of treatment, the number of CRM performed was 2 sessions in 21/147 patients (14%), 3 sessions in 48 patients (33%), 4 sessions in 51 patients (35%), 5 sessions in 17 patients (12%), and 6 sessions in 10 patients (6%), with a mean of 3.6 (SD 1.0).

Preclinic YouTube searching was reported by 36/147 patients (women: n=28, 19%; men: n=8, 6%), and preclinic self-treatment was reported by 11 patients (women: n=9, 7%; men: n=2, 1%). Postclinic YouTube searching was reported by 21 patients (women: n=14, 10%; men: n=7, 5%), and postclinic self-treatment was reported by 5 patients (women: n=4, 3%; men: n=1, 1%).

In the gender-stratified GEE repeated measures analysis of YouTube searching behavior, women showed significantly higher odds of searching before the clinic visit compared to after (odds ratio [OR] 2.389, 95% CI 1.195‐4.778; *P*=.01) (Table 1).

**Table 1.** Gender-stratified generalized estimating equations results for preclinic versus postclinic YouTube searching.

Parameter	β(SE)	Hypothesis test		Odds ratio	95% Wald CI	
		Wald Chi-square^a^	*P* value		Lower	Upper
Women						
Chronic disease^b^	–0.530 (0.4316)	1.508	.22	0.589	0.253	1.372
Age	–0.003 (0.0123)	0.067	.80	0.997	0.973	1.021
Preclinic^c^	0.871 (0.3535)	6.071	.01	2.389	1.195	4.778
Men						
Chronic disease	1.038 (0.8968)	1.339	.25	2.823	0.487	16.370
Age	–0.026 (0.0231)	1.239	.27	0.975	0.932	1.020
Preclinic	0.353 (0.4955)	0.509	.48	1.424	0.539	3.761

a All degrees of freedom are 1.

b Chronic disease: diabetes or hypertension

c Preclinic: before visiting the clinic.

In the gender-stratified GEE analysis of self-treatment behavior, women with chronic disease had significantly lower odds of self-treatment compared to those without chronic disease (OR 0.125, 95% CI 0.016‐0.976; *P*=.047) (Table 2).

**Table 2.** Gender-stratified generalized estimating equations results for preclinic versus postclinic self-treatment.

Parameter	β (SE)	Hypothesis test		Odds ratio	95% Wald CI	
		Wald Chi-square^a^	*P* value		Lower	Upper
Women						
Chronic disease^b^	−0.530 (0.4316)	3.934	.047	0.125	0.016	0.976
Age	−0.003 (0.0123)	0.648	.42	1.010	0.985	1.036
Preclinic^c^	0.871 (0.3535)	2.214	.14	2.417	0.756	7.727
Men						
Chronic disease	1.038 (0.8968)	1.384	.24	10.711	0.206	556.192
Age	−0.026 (0.0231)	2.691	.10	0.945	0.884	1.011
Preclinic	0.353 (0.4955)	0.329	.57	2.090	0.169	25.895

a All degrees of freedom are 1.

b Chronic disease: diabetes or hypertension.

c Preclinic: before visiting the clinic.

Negative binomial regression analysis evaluating the impact of self-treatment status (preclinic or postclinic) on the number of CRM sessions revealed no statistically significant association (*P*=.92 and *P*=.97, respectively) (Table 3).

**Table 3.** Negative binomial regression results evaluating the impact of self-treatment timing on the number of canalith repositioning maneuvers.

Parameter	β (SE)	Hypothesis test		Odds ratio
		Wald Chi-square (*df*)	*P* value	
Preclinic^a^	0.038 (0.3567)	0.011 (1)	.92	1.038
Postclinic^b^	−0.019 (0.5188)	0.001 (1)	.97	0.981

a Preclinic: before visiting the clinic.

b Postclinic: during the treatment period.

Table 4 presents an overview of the included studies by author, origin country, publication type, research focus, and primary topic area. This summary provides a contextual understanding of the existing literature on BPPV, YouTube-based health information, and related digital health research.

**Table 4.** Descriptive characteristics of included studies.

No.	Author (year)	Country	Publication type	Focus area	Main topic / Summary
1	Grajales FJ et al (2014) [1]	USA	Journal article	Medical Education	Overview of social media applications in health care
2	Madathil KC et al (2015) [2]	USA	Journal article	Digital Health / Information Quality	YouTube as a source of health information
3	Rhim GI (2018) [3]	Korea	Journal article	Clinical	Serum vitamin D and long-term outcomes in BPPV^a^
4	Dix MR et al (1952) [4]	UK	Journal article	Clinical	Foundational study on vestibular system pathology
5	McClure JA (1985) [5]	Canada	Journal article	Clinical	Description of horizontal canal BPPV
6	Bhattacharyya N et al (2017) [6]	USA	Journal article	Clinical Practice	clinical guideline for BPPV
7	Kerber KA et al (2012) [7]	USA	Journal article	Digital Health / Education	Validity of Epley maneuver videos on YouTube
8	Kim HJ et al (2020) [8]	Korea	Journal article	Clinical Epidemiology	Etiologic distribution of dizziness
9	Delli K et al (2016) [9]	Netherlands	Journal article	Digital Health / Information Quality	YouTube as a source of information
10	Biggs TC et al (2013) [10]	UK	Journal article	Digital Health / Public Education	YouTube information quality on rhinosinusitis
11	Hassona Y et al (2016) [11]	Jordan	Journal article	Digital Health / Information	YouTube as a source of oral cancer information
12	Landrum AR (2021) [12]	USA	Journal article	Science Communication	Gender differences in science audiences on YouTube
13	Langford AT et al (2022) [13]	USA	Journal article	Digital Health / Information	YouTube use among patients with chronic illness
14	Lim MSC et al (2022) [14]	Australia	Journal article	Digital Health / Behavior	Social media use for health information among young adults
15	Khan ML. (2017) [15]	USA	Journal article	Communication / Behavioral Science	Motivations for user engagement on YouTube
16	Moll R et al (2017) [16]	Canada	Journal article	Science Education	Development of social media and science learning survey
17	Naslund JA et al.\ (2014) [17]	USA	Journal article	Mental Health / Social Support	Peer support via YouTube for individuals with mental illness
18	Bujnowska-Fedak MM et al (2020) [18]	Poland	Journal article	Digital Health / Behavior	Impact of online health information on patient decisions
19	Mohamed F et al (2024) [19]	UAE	Journal article	Digital Health / Decision Tool	User experience with health-related YouTube content
20	Kerber KA et al (2022) [20]	USA	Journal article	Behavioral Intervention	Development and evaluation of instructional video for BPPV self-management
21	Kim HJ et al (2023) [21]	Korea	Journal article	Clinical Trial	Effect of self-treatment for recurrent BPPV
22	Liu T et al (2025) [22]	China	Journal article	Digital Health / Social Learning Theory	Five-dimensional extension of Social Learning Theory
23	Lau Y et al (2025) [23]	Singapore	Journal article	AI-Based^b^ Psychotherapy	Therapeutic efficacy of AI-based interventions

a BPPV: benign paroxysmal positional vertigo.

b AI: artificial intelligence.

## Discussion

### Principal Results

Social media, including YouTube, are dynamic and interactive computer-mediated communication tools with high penetration rates in the general population in high-income and middle-income countries. However, in medicine and health care, many stakeholders remain unaware of social media’s relevance [1]. CRMs, the standard treatment for BPPV, typically take about 10‐15 minutes to perform. Because of the procedural complexity, patients cannot accurately perform self-treatment based on short-form content or brief blog posts. Currently, YouTube is the only widely accessible platform that allows patients to visually observe and replicate these maneuvers. Therefore, almost all previous studies investigating online resources related to BPPV have focused exclusively on YouTube as the primary platform. Analyzing the overall results of YouTube content related to health reveals several key issues. Much of the information is misleading, with a high probability that consumers will encounter such content during their searches [2]. Posts created by professional organizations are more reliable. In contrast, videos uploaded by individual users are less useful [9,10]. Additionally, there is little difference in view counts between misleading and accurate posts. Most studies have focused on evaluating the validity of the information, classifying it as valid, erroneous, or anecdotal [11].

Public media serves as an avenue for informal science learning, and women may engage with YouTube content differently than men, viewing it more as an educational resource. Women are often motivated by practical or instrumental needs rather than by pure curiosity [12]. Our study found that women’s YouTube searching behavior significantly decreased after visiting the clinic, unlike men, whose search rates remained nearly unchanged. Specifically, the proportion of women engaging in YouTube searching was reduced by half following their clinic visit, suggesting that clinical consultation may have a stronger impact on modifying information-seeking behavior among women.

Previous studies have reported that approximately 30%‐35% of adults with chronic conditions such as hypertension and diabetes use YouTube to watch health-related videos [13]. In our study, despite BPPV being an acute condition, the preclinic YouTube searching rate was 25%, suggesting that patients with acute dizziness symptoms are also highly likely to seek online information before clinic visits. This relatively high level of previsit searching underscores the need for clinicians to address online health information use during consultations.

Patients often find the video format on YouTube easy to learn from and tend to evaluate the accuracy of health information by cross-checking multiple videos or judging the credibility of presenters [14].

Previous research has identified several motivations for YouTube use, such as information-seeking, social interaction, and entertainment [15,16]. Studies have also documented gender differences in YouTube usage patterns. Generally, men are more likely to watch educational or science-related content, whereas women more frequently view health and wellness videos. Moreover, the motivations for engaging with science-related content differ by gender—men more often seek entertainment or general information, while women tend to use such videos for learning or practical purposes [12]. In line with these previous findings, our study also showed that women were more likely than men to use YouTube videos as a tool for self-treatment related to BPPV.

People are increasingly using YouTube as a platform to seek support from others with similar health conditions, find answers to health-related questions, and gather information before planned medical procedures [17]. The impact of YouTube on users’ health-related behaviors and decisions is substantial; more than 50% of surveyed users reported that online health-related content influenced their decisions regarding health [18].

According to a recent study by Mohamed et al, 87.6% (n=2630) of participants watched health-related content on YouTube, and 84.7% (n=2542) reported making decisions based on the content viewed. Among users, 40% watched YouTube videos to decide whether to consult a physician or adopt a specific health-related practice [19]. In this study, women with chronic disease showed significantly lower odds of attempting self-treatment. This suggests that these patients may be more cautious about adopting unsupervised online practices and more likely to rely on professional guidance.

Research on BPPV and YouTube includes studies that evaluated the accuracy of videos on the treatment of posterior canal BPPV using the Epley maneuver. In one study, 21 out of 33 videos were found to be valid and potentially useful for patient home treatment or educational purposes [7]. Other studies have focused on evaluating educational videos for treating posterior canal BPPV following a physician’s diagnosis and providing a web-based diagnosis and treatment for BPPV in cases of symptom recurrence [20,21]. While these studies focused on the validity and accuracy of YouTube or video content, our study is distinct in that it adopts a user-centered approach.

However, this study found that prior self-treatment did not significantly affect the number of in-clinic CRM sessions required, suggesting that while inappropriate self-treatment may complicate diagnosis, it does not necessarily increase the overall treatment burden when managed in a clinical setting.

According to Liu et al [22], digital health behavior among older adults was conceptualized by extending Social Learning Theory into five dimensions. While our study focuses on actual clinical and behavioral aspects, both studies intersect in the mechanism of self-learning through observation. In particular, the observational learning dimension corresponds to visual imitation and behavioral acquisition through watching YouTube videos, whereas self-efficacy relates to patients’ confidence and belief that they can perform self-treatment independently after viewing such instructional content [22].

Lau et al [23] conducted a meta-analysis that quantitatively verified the therapeutic efficacy of artificial intelligence, AI-based psychotherapeutic interventions. In the future, the findings of our study may help overcome the limitations and risks associated with nonprofessional self-treatment and contribute to the development of AI-assisted approaches for the management of BPPV [23].

### Limitations

This study has several limitations. Because the questionnaire was administered after treatment, there is a potential for recall bias, as some participants may have inaccurately reported their prior exposure to YouTube or other online resources. There is a possibility of a type II error in the study sample. Patients whose symptoms improved through self-treatment using YouTube videos might not have visited the hospital, thereby being excluded from the survey and potentially affecting the study results. Additionally, this study’s relatively small sample size did not allow for an investigation of other sociodemographic factors, such as occupation and education level, and their association with YouTube usage.

### Conclusion

Our findings show that clinical consultation leads to meaningful changes in YouTube searching and self-treatment behaviors for BPPV, especially among women. While prior self-treatment attempts did not increase the number of CRM sessions needed, our findings underscore the importance of addressing online health information use and supporting patient education during clinical care.

## Abbreviations

AI: artificial intelligence
BPPV: benign paroxysmal positional vertigo
CRM: canalith repositioning maneuver
GEE: Generalized Estimating Equations
OR: odds ratio
